# Do Kombucha Symbiotic Cultures of Bacteria and Yeast Affect Bacterial Cellulose Yield in Molasses?

**DOI:** 10.3390/jof7090705

**Published:** 2021-08-28

**Authors:** Putu Virgina Partha Devanthi, Katherine Kho, Rizky Nurdiansyah, Arnaud Briot, Mohammad J. Taherzadeh, Solmaz Aslanzadeh

**Affiliations:** 1 Indonesia International Institute for Life Sciences, Pulomas Barat Kavling 88, Jakarta 13210, Indonesia; katherine.k@i3l.ac.id (K.K.); rizky.nurdiansyah@i3l.ac.id (R.N.); 2 Bioprocess & Process Engineering Department, Polytech Nantes, University of Nantes, Gavy Océanis, CS 70152, 44633 Saint-Nazaire, France; arnaud.briot@etu.univ-nantes.fr; 3 Swedish Centre for Resource Recovery, University of Borås, 50190 Borås, Sweden; mohammad.taherzadeh@hb.se

**Keywords:** *Komagataeibacter intermedius*, *Dekkera bruxellensis*, co-culture, bacterial cellulose, molasses

## Abstract

Bacterial cellulose (BC) is a valuable biopolymer typically observed in Kombucha with many potential food applications. Many studies highlight yeast’s roles in providing reducing sugars, used by the bacteria to grow and produce BC. However, whether yeast could enhance the BC yields remains unclear. This study investigates the effect of yeast *Dekkera bruxellensis* on bacteria *Komagataeibacter intermedius* growth and BC production in molasses medium. The results showed that the co-culture stimulated *K. intermedius* by ~2 log CFU/mL, which could be attributed to enhanced reducing sugar utilization. However, BC yields decreased by ~24%, suggesting a negative impact of *D. bruxellensis* on BC production. In contrast to other studies, regardless of *D. bruxellensis*, *K. intermedius* increased the pH to ~9.0, favoring the BC production. Furthermore, pH increase was slower in co-culture as compared to single culture cultivation, which could be the reason for lower BC yields. This study indicates that co-culture could promote synergistic growth but results in the BC yield reduction. This knowledge can help design a more controlled fermentation process for optimum bacterial growth and, ultimately, BC production.

## 1. Introduction

Bacterial cellulose (BC) is an extracellular polysaccharide biofilm matrix synthesized by specific types of bacteria. Unlike plant-derived cellulose, BC has a high purity level, as it does not contain lignin, pectin, and hemicellulose. Although BC structure is similar to plant cellulose, it possesses several unique characteristics, including higher crystallinity, Young’s modulus, tensile strength, thermal stability, elasticity, and porosity [1,2]. Furthermore, its large surface area increases its water holding capacity and its ability to form strong bonding with other biomaterials, enzymes, and nanoparticles [3]. These features make BC an attractive biopolymer for many applications, especially in food industries [4]. One of the most popular food products derived from BC is known as *nata-de-coco*, produced by *Acetobacter xylinum* grown in coconut water [5]. BC also finds promising applications as fat replacer [6,7], texture modifier [8], probiotic and enzyme encapsulating material [9,10], as well as film for food coating and packaging [11,12].

Despite all the unique properties, BC’s high production cost still limits its production at an industrial scale. BC production requires a fermentation medium rich in glucose and other nutrients, which is costly and can account for up to 30% of the total production cost [13]. Low BC yield and long fermentation time also contribute to the high production cost. Therefore, an alternative cheap fermentation media and suitable microbial cultures are paramount in low-cost BC production at an industrial scale. In recent years, sugarcane molasses, a by-product of a sugar refinery, has attracted broad interest as a cheaper alternative fermentation media for BC production. Molasses is known for its high amounts of fermentable sugars, such as fructose, sucrose, glucose, and nutrients such as nitrogen and vitamins, essential for BC formation. However, BC-producing bacteria’s ability to break down sucrose, which constitutes 50% of sugars in molasses, is limited, resulting in lower BC yield. Consequently, expensive pre-treatments would be required, such as acid, heat, or enzymatic treatment [1], to hydrolyse the sucrose before the fermentation process, ultimately leading to higher production costs.

BC biosynthesis is typically observed during the production of Kombucha, a beverage obtained by fermenting tea and sugar using a symbiotic culture of yeast and acetic acid bacteria. The synergistic interactions between these two microbial groups have been frequently highlighted in the literature [14,15,16,17]. Yeast is known as the invertase producer that breaks down sucrose into reducing sugars, increasing their availability to yeast and acetic acid bacteria. These reducing sugars are then metabolized by yeast and acetic acid bacteria into ethanol and organic acids. Glucose, the building block for BC biosynthesis, is also used by the acetic acid bacteria to produce BC, which is observed as a floating pellicle on Kombucha tea’s surface [14]. Although it is tempting to infer that yeast and acetic acid bacteria co-culture could increase the BC yield, the study supporting this claim is still limited. The yeast-acetic acid bacteria synergistic interactions described above were only reported in the context of Kombucha fermentation, using sucrose as the sole carbon source, with organic acids as the main product of interest [17,18]. Moreover, current literature reporting the optimization of BC production only focus on using acetic acid bacteria in a single culture instead of a co-culture system with yeast [1,2]. Therefore, this study investigates the interaction between Kombucha-derived yeast and acetic acid bacteria in a molasses medium by monitoring growth, pH changes, and reducing sugar utilization. Since yeast was expected to increase the availability of glucose that serves as a BC building block, the impact of yeast inoculation on BC yield was also monitored.

## 2. Materials and Methods

### 2.1. Materials

Kombucha culture was provided by PT Tujju Kombucha, Indonesia. The culture was stored at 4 °C until use. Molasses was supplied by PT Andalan Furnindo, Indonesia, with characteristics presented in Table 1. Black tea (Teh Perbawati Goalpara, Sukabumi, Indonesia), sugar (Gulaku Murni, Indonesia), and pure caffeine (PureBulk, Roseburg, OR, USA) were purchased from the local market in Jakarta. The pH of molasses was maintained by adding acetate buffer prepared using acetic acid (Merck, Darmstadt, Germany) and sodium acetate (Merck, Darmstadt, Germany). Phosphate Buffer Saline (PBS; Sigma-Aldrich, St. Louis, MI, USA) was used for serial dilution of microbial cells. Microbiological growth media used were Nutrient Agar (NA; Merck, Darmstadt, Germany), Potato Dextrose Agar (PDA; HiMedia, Mumbai, India), and Hestrin and Schramm (HS) comprised of 20 g/L glucose, 5 g/L peptone, 5 g/L yeast extract, 2.7 g/L Na_2_HPO_4_, and 1.15 g/L citric acid. The bacteria and yeast growth were controlled by supplementing the agar media with chloramphenicol (HiMedia, Mumbai, India) and cycloheximide (Sigma-Aldrich, St. Louis, MI, USA), respectively.

### 2.2. Methods

#### 2.2.1. Kombucha Culture Adaptation in Molasses Medium

Prior to the isolation step, the Kombucha culture was adapted in the molasses medium to ensure that only the microbes capable of growing and producing BC in molasses were isolated. The adaptation was carried out gradually in three steps using three different media: (A) 100% sweetened tea, (B) 50% sweetened tea and 50% molasses, and (C) %100 molasses. Medium A was prepared by boiling 10 g of black tea and 100 g of table sugar in 1 L of type III water. Medium C was prepared by mixing 100 g of molasses with 1 L of acetate buffer solution (200 mM, pH 4.75). Medium B was prepared by combining medium A and C to a ratio of 1:1. Before use, each medium was filtered through filter paper 125 mm ∅ (cat no 1004 125 Whatman) and autoclaved at 121 °C for 15 min.

The adaptation step started by first transferring the Kombucha culture (30 mL) to medium A (300 mL) and incubated at 30 °C for 7 days until the BC layer was observed. Then, the culture from medium A (50 mL) was transferred to 500 mL of medium B. After 5 days of incubation, the culture from medium B (50 mL) was transferred to 500 mL of medium C and incubated for 6 days at the same temperature.

#### 2.2.2. Microbial Isolation from Adapted Kombucha Culture

Bacteria and yeast were isolated from both liquid and solid phases (BC). Liquid (1 mL) and BC (1 g) were collected and serially diluted using PBS before culturing it on their respective agar media. Bacteria were isolated using NA and HS agar (20 g/L glucose, 5 g/L peptone, 5 g/L yeast extract, 2.7 g/L Na_2_HPO_4_, 1.15 g/L citric acid, and 15 g/L agar) supplemented with 0.5 mg/mL cycloheximide. Meanwhile, yeast was isolated using PDA supplemented with 25 mg/mL chloramphenicol. The agar plates were incubated at 30 °C for 2–3 days. The dominant colonies were picked from the agar with the highest dilution and subjected to several purification steps by streaking on the same agar media used during isolation. The bacterial cells were Gram-stained before microscopic evaluation, while yeast cells were observed without staining. The pure isolates were cryopreserved in 1.5 mL of 20% glycerol solution and kept at −80 °C until use.

#### 2.2.3. Screening for BC-Producing Capacity

Five bacterial isolates (13.7.KBC.HSA, 13.7.KBC.HSB, 13.7.KBC.HSC, 13.7.KBC.HSD, and 13.7.KBC.HSE) obtained from the previous step were screened for their ability to produce BC. The frozen stocks were revived by plating on HS agar, followed by incubation at 30 °C for 7 days. After a week, a loopful of each bacterial isolate was transferred to 25 mL of HS broth and incubated at 30 °C for 5 days under static conditions. After 5 days, each isolate’s optical density (OD) was measured at 600 nm and adjusted to ~0.2, which further served as an inoculum source. The inoculum (1 mL) was then transferred to each well of 6-well plates containing 9 mL of molasses medium, comprising 150 g/L molasses (70.38 g/L sucrose and 76.14 g/L reducing sugars) and 500 mg/L caffeine in acetate buffer (200 mM, pH 4.75). After 14 days of incubation at 30 °C under static conditions, the BC yield was examined, and the isolate with the highest BC producing capacity was selected for further study.

#### 2.2.4. Identification of Bacterial and Yeast Isolates by DNA Sequencing

Bacteria and yeast isolates were identified by sequencing the 16S rRNA gene and the D1/D2 region of the 26S rRNA gene, respectively. The DNA template was prepared by gene amplification using the colony PCR technique. The 16S rRNA gene was amplified using universal primers (27F 5′-AGAGTTTGATCCTGGCTCAG-3′ and 1492R 5′-GGTTACCTTGTTACGACTT-3′), while the D1/D2 region was amplified using NL1 (5′-GCATATCAATAAGCGGAGGA AAAG-3′) and NL4 (5′-GGTCCGTGTTTCAAGACGG-3′) primers. PCR reactions for 16S rRNA gene amplification were performed under the following conditions: 5 min initial denaturation at 95 °C, 34 cycles of denaturation at 94 °C for 1 min, annealing at 55 °C for 1 min, extension 72 °C for 2 min, and final extension at 72 °C for 5 min. The PCR conditions used for D1/D2 region amplification were as follows: 10 min initial denaturation at 94 °C, 35 cycles of denaturation at 94 °C for 1 min, annealing at 52 °C for 1 min, extension at 72 °C for 2 min, and final extension at 72 °C for 10 min. The PCR product was analyzed using electrophoresis with 1% *w*/*v* agarose gel in Tris-borate-EDTA. Agarose gel was then soaked in ethidium bromide (EtBr) and visualized under UV light. Then, the PCR products were sent to Macrogen (Seoul, Korea) for further purification and sequencing. The sequence similarity was then determined using the BLASTn program in the GenBank database (https://www.ncbi.nlm.nih.gov/) (accessed on 15 January 2020). The gathered sequences were used to reconstruct a neighbor joining tree (500 bootstraps) with close and distant-related species based on the BLASTn result using MEGAX software [19]. The distant-related species were selected based on identified fungi and bacteria in Kombucha to maintain relevance and some reference sequences [16].

#### 2.2.5. Bacteria and Yeast Interactions in Molasses Medium

The selected BC producing bacteria and yeast interactions were evaluated by comparing their growth in single and co-culture. The experiment was conducted in parallel in several 6-well plates. Each well contained 10 mL of molasses medium containing 150 g/L molasses and 500 mg/L caffeine dissolved in acetate buffer (200 mM, pH 4.75). Meanwhile, the inoculum of the selected BC producing bacteria and yeast was cultured in HS and PDB liquid media, respectively, for 4 days under a static condition at 30 °C. Different inocula (5% *v*/*v*, 10% *v*/*v*, and 15% *v*/*v*) were then added to each well. In the case of co-culture, bacteria and yeast inoculum were combined at a ratio of 1:1. The fermentation was conducted in a static condition at 30 °C for 15 days. This experiment was repeated on two separate occasions.

#### 2.2.6. Bacterial and Yeast Cell Enumeration

Cell enumeration was carried out by taking samples of 0.1 mL, followed by serial dilution in PBS and plating on HS supplemented with cycloheximide and PDA supplemented with chloramphenicol for bacteria and yeast, respectively. Bacteria and yeast colonies were enumerated after 2–3 days of incubation at 30 °C.

Specific growth rate μ (h^−1^) were calculated by Equation (1) [20]

(1) $$\mu =\;\frac{\mathrm{Ln}(X_{0}-X_{t})\;}{t-t_{0}}$$

where in:

X_t_ and X_0_ are the microbial population (CFU mL^−1^) at t and initial time, respectively

t and t_0_ are the t and initial time when the sample is measured, respectively

μ is specific growth rate (1 h^−1^)

#### 2.2.7. BC Yield Measurement

After the fermentation, the BC was collected and oven-dried at 105 °C for 20 h. The weight measurement was carried out using an analytical balance. To ensure the water was removed entirely, this process was repeated until the stable weight was achieved. The BC yield is expressed as the BC dry weight per volume of the fermentation medium (g/L).

#### 2.2.8. pH and Reducing Sugar Measurement

The pH of the medium was measured using a pH meter. Sugar consumption was monitored by calculating the remaining reducing sugar using the dinitrosalicylic acid (DNS) method. The sample was diluted 100× with Type III water. Then, 2 mL of sample was mixed with 1 mL of 3,5-dinitrosalicylic acid reagent using a vortex. The mixture was boiled for 15 min and then cooled down in an ice bath before 9 mL of Type III water was added. The absorbance was then read at 540 nm.

#### 2.2.9. Statistical Analysis

Significant differences among means were tested by one-way analysis of variances (ANOVA) using XLSTAT™ version 2020.5.1 (Addinsoft, New York, NY, USA) at *p* < 0.05 and Tukey’s test was applied for means comparison.

## 3. Results and Discussion

### 3.1. Screening and Identification of Potential BC Producing Bacteria and Predominant Yeast

A total of 6 isolates, consisting of 1 yeast (13.7.KBC.HSF) and 5 bacterial isolates (13.7.KBC.HSA, 13.7.KBC.HSB, 13.7.KBC.HSC, 13.7.KBC.HSD, and 13.7.KBC.HSE) were obtained from the Kombucha culture adapted in molasses medium. All bacterial isolates were screened for their BC producing capacity in molasses medium. The results showed that after 14 days, bacteria isolates 13.7.KBC.HSA and 13.7.KBC.HSB produced 0.018 g/L and 0.019 g/L of BC, respectively, which were the highest among the isolates. Although both isolates produced a similar yield, the BC produced by 13.7.KBC.HSB was more brittle. Therefore, 13.7.KBC.HSA was selected as a starter culture for the experiment.

**Figure 1:**
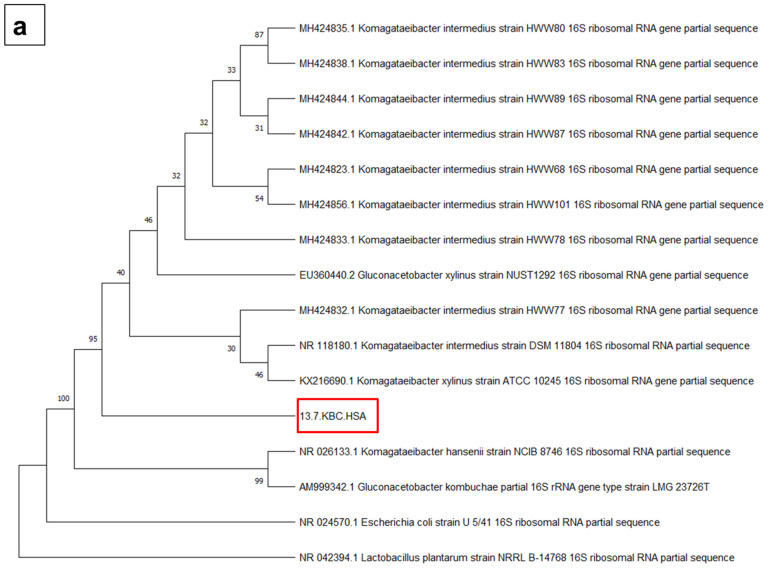

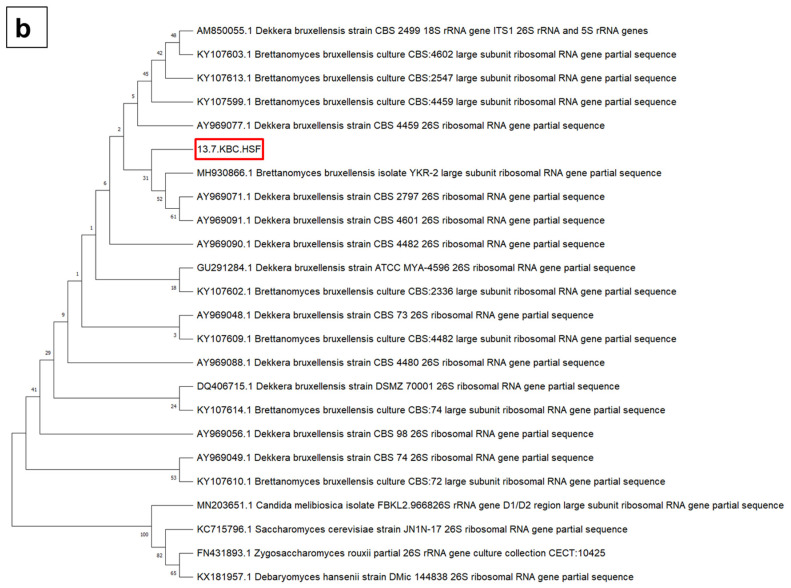
Neighbor-Joining tree of the sequencing result based after BLAST analysis. (**a**) Bacterial isolate’s 16S rRNA sequence; (**b**) yeast isolate’s 26S rRNA sequence with closely identical sequences and several distant species. The tree was reconstructed using 500 bootstrapping. Red box (**a**) 13.7.KBC.HSA indicates the bacterial isolate and (**b**) 13.7.KBC.HSF indicates yeast isolate from the Kombucha culture.

Isolate 13.7.KBC.HSA was subsequently identified through 16S rRNA gene sequencing, and the result showed a 99.91% similarity with the sequence of *Komagataeibacter intermedius* (Figure 1a). *K. intermedius* is a Gram-negative rod-shaped bacteria commonly found in fruit juice, wine vinegar, and Kombucha [2,21]. Gaggìa et al. [22] reported the presence of *K. intermedius* and *Komagateibacter* spp. in Kombucha regardless of the tea types used for the fermentation (black tea, green tea, or rooibos tea). The bacteria is well-known for its ability to produce BC with mannitol as the sole carbon source. According to Fernández et al. [2], the *K. intermedius* strain isolated from a commercial wine vinegar showed 48% higher BC producing ability than *K. xylinus*, a commonly used BC producing bacteria. The BC produced was also free from impurities, exhibited a high crystallinity index, and showed similar mechanical properties to the one produced by *K. xylinus.* Unlike *K. xylinus*, *K. intermedius* is able to produce BC within a wide pH range (4–9), and maximum production can occur within a short period in alkaline conditions [21]. In a study by Tyagi and Suresh [1], *K. intermedius* demonstrated the ability to produce BC in molasses medium; however, the yield was reduced by ~60% compared to those obtained using pure glucose or fructose medium.

The most dominant yeast isolated (13.7.KBC.HSF) was identified through the D1/D2 region of the 26S rRNA gene sequencing and was shown to have 99.83% similarity with the sequence of *Brettanomyces/Dekkera bruxellensis* (Figure 1b). *D. bruxellensis* is one of the yeast species that is commonly found in Kombucha [23,24] and other fermented products, such as beer [25], wine [26], cider [27], and kefir [28]. This yeast has a complex phenotypic, genotypic, and population structure, which can vary based on the substrate, isolation origin, and geographical origin, suggesting the anthropic influence of the species’ diversity [29]. *D. bruxellensis* has been considered industrially important due to its high resistance to osmotic and ethanol stress, the ability to grow in oxygen-limited environments, and low pH. It also demonstrates the ability to produce biofilm with characteristics known to be strain-dependent [30]. The genetic sequence analysis of different *D. bruxellensis* strains reveals that they are equipped with a gene cluster comprising of a nitrate transporter, nitrate reductase, nitrite reductase, and two Zn(II)_2_ Cys_6_ type transcription factors that enable the utilization of nitrate as a sole nitrogen source [31,32,33]. Such a cluster might allow the yeast to thrive in low-nitrogen environments like molasses. *D. bruxellensis* is also capable of utilizing sucrose efficiently due to the expression of a high-efficiency sucrose transporter, which allows *D. bruxellensis* to outcompete *Saccharomyces cerevisiae* in sucrose-based fermentations [34,35].

### 3.2. Synergistic Growth of K. intermedius and D. bruxellensis in Co-Culture

As two or more microbial species co-exist, a mutualistic or antagonistic interaction may occur since each microbe requires different nutrition and optimal growing conditions, which lead to different growth rates. To find out which types of interactions occur between *K. intermedius* and *D. bruxellensis* in the co-culture system, the population of both species in single and co-culture was compared.

**Figure 2:**
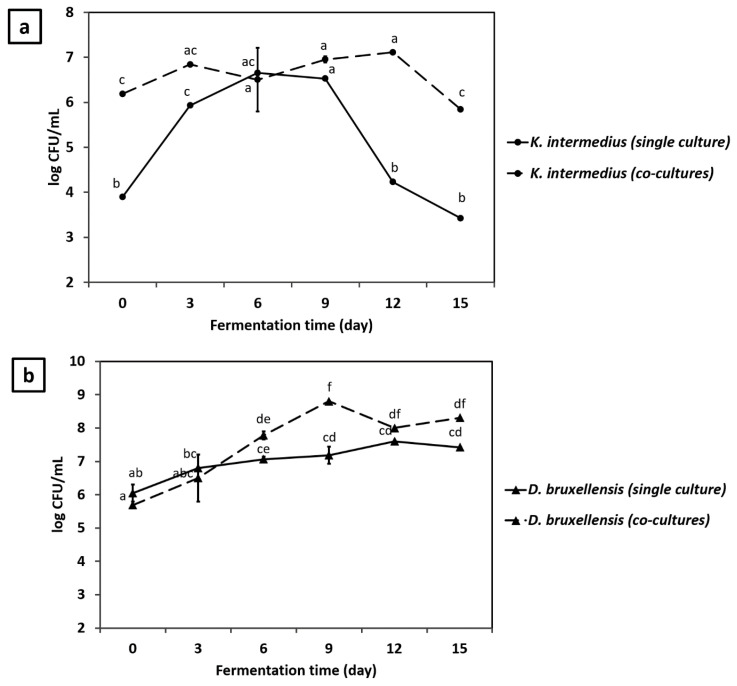
The growth of (**a**) *K. intermedius* and (**b**) *D. bruxellensis* as single and co-culture. Culture conditions: media composition, 150 g/L molasses, 500 mg/L caffeine, acetate buffer (200 mM, pH 4.75); inoculum size, 10% *v*/*v*; *K. intermedius*: *D. bruxellensis* ratio, 1:1; temperature, 30 °C. Means with different letters are significantly different (*p* < 0.05).

During the first 3 days of single culture fermentation, *K. intermedius* multiplied at a specific growth rate of 0.188 ± 0.01 h^−1^, which increased by 14.4% in co-culture to 0.215 ± 0.01 h^−1^, suggesting a stimulatory effect of *D. bruxellensis* on *K. intermedius* growth (Table 2). In co-culture, *K. intermedius* maintained a relatively stable population during the first 12 days before it was decreased from 7.11 log CFU/mL to 5.85 log CFU/mL on day 15. By contrast, when *D. bruxellensis* was absent, *K. intermedius* population started to decrease on day 9, from 6.52 log CFU/mL to 3.42 log CFU/mL on day 15 (Figure 2a).

A similar stimulatory effect of co-culture was also observed on *D. bruxellensis*, indicated by an increase in specific growth rate by 4.69% from 0.213 ± 0.18 h^−1^ to 0.223 ± 0.01 h^−1^ during the first 3 days of fermentation (Table 2). In co-culture, *D. bruxellensis* constantly multiplied during the first 9 days, increasing its population from 5.68 log CFU/mL to 8.80 log CFU/mL. While during the same period, *D. bruxellensis* in single culture propagated slower, increasing its population only by ~1 log CFU/mL from 6.05 log CFU/mL to 7.18 log CFU/mL (Figure 2b). Similar synergistic growth has also been observed between other bacterial and yeast species involved in soy sauce [36] and milk [37] fermentation. Such synergistic interaction could be associated with the production of metabolites such as pyruvate, amino acids, and vitamins, essential for one’s population growth. In addition, the transformation of potential inhibitory substances into other compounds that are less toxic to one’s population growth could also explain such synergistic interaction [38].

### 3.3. The Effect of Co-Culture on Reducing Sugar Content

Yeast has been known as an important invertase producer that breaks down sucrose into reducing sugars, which are more accessible to any microbial members of the Kombucha consortium. BC-producing bacteria will subsequently use these reducing sugars to produce organic acids and BC as a floating pellicle. In this study, the reducing sugar consumption rates by *K. intermedius* and *D. bruxellensis* in both single and co-culture were monitored.

**Figure 3:**
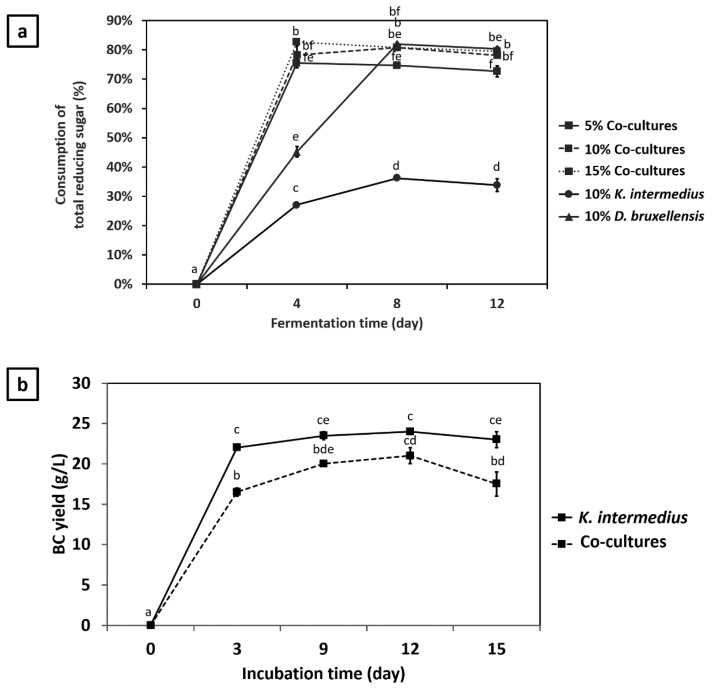

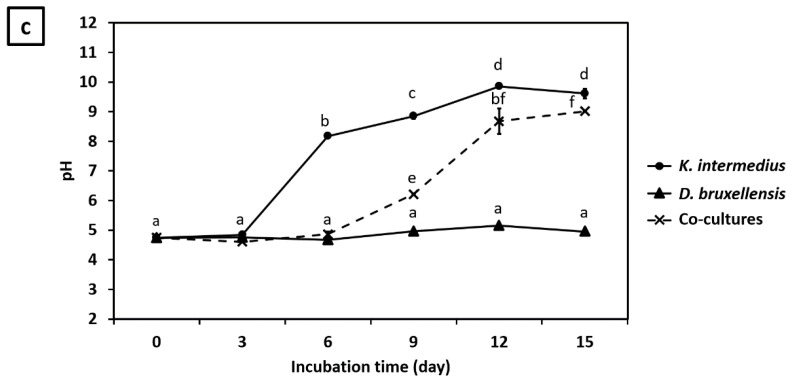
(**a**) Reducing sugar consumption by single and co-culture varying in inoculum sizes (5%, 10%, 15% *v*/*v*); (**b**) BC yield and (**c**) pH changes during single and co-culture fermentation using inoculum size of 10% *v*/*v*. Culture conditions: media composition, 150 g/L molasses, 500 mg/L caffeine, acetate buffer (200 mM, pH 4.75); *K. intermedius*: *D. bruxellensis* ratio, 1:1; temperature, 30 °C. Means with different letters are significantly different (*p* < 0.05).

As shown in Figure 3a, maximum reducing sugar consumption by *K. intermedius* and *D. bruxellensis* in single culture occurred on day 8 followed by a plateau. However, the maximum amount of reducing sugars consumed by *K. intermedius* only reached 36%, while *D. bruxellensis* reached up to 82%, leaving ~50 g/L and ~14 g/L reducing sugars in the media, respectively. The results confirm that *D. bruxellensis* could consume higher reducing sugar than *K. intermedius*, which might explain its survival during fermentation (Figure 2a). A higher reducing sugar consumption rate by *D. bruxellensis* was expected since it can break down sucrose into reducing sugars, increasing their availability to both microbes. However, the increase in reducing sugar concentration could not be observed, suggesting that the consumption rate exceeded the production rate, resulting in a constant decrease in reducing sugar concentration during the fermentation.

The reducing sugar consumption rates of *K. intermedius* and *D. bruxellensis* in single and co-culture during the first 4 days of fermentation were calculated and compared as presented in Table 2. In single culture, the sugar consumption rate was 62.3% higher for *D. bruxellensis* as compared to *K.* *intermedius*, which was about 25.8 ± 2 g/L/day and 15.9 ± 0.01 g/L/day, respectively. The consumption rate for *D. bruxellensis* and *K. intermedius* increased in co-culture by 79.8% and 191%, respectively, to 46.4 ± 3.2 g/L/day, slightly higher than the sum of their individual consumption rate. Such an increased consumption rate caused the depletion of nearly 80% of total reducing sugar in co-culture within the first 4 days of fermentation (Figure 3a). The result was in agreement with previous studies reporting the importance *D. bruxellensis* to make sugars more available to *K. intermedius* [14,16,17].

### 3.4. The Effect of Co-Culture on pH Changes

In single culture, *K. intermedius* caused a significant pH increase from 4.74 to 9.61, while *D. bruxellensis* maintained a constant pH (~4.8) throughout fermentation. On the other hand, the pH increased more slowly in co-culture, as it took 12 days for the co-culture to reach pH > 8.0, whereas it took only 6 days for the single culture. This observation could be attributed to *D. bruxellensis’* ability to produce acetic acid [39]. However, pH increase was unexpected with the presence of *K. intermedius* in both single and co-culture, since acetic acid bacteria of the *Komagataeibacter* genus are well known for their ability to produce various organic acids, such as acetic acid, gluconic acid, and glucuronic acid causing the pH to decrease. Although acid production is less significant than *K. xylinus*, a decrease in pH was previously observed by Fernández et al. [2]. Most investigations performed on BC production by *K. intermedius* have reported pH reduction throughout fermentation. For instance, Lin et al. [21] conducted a fermentation using *K. intermedius* FST213-1 in an HS medium with an initial pH of 8.0 and found the pH to drop to ~4.0 after 4 days, which remained constant until the end of the fermentation. Furthermore, Fernández et al. [2] reported a pH reduction by one order of magnitude within 96 h when *K. intermedius* JF2 was cultivated in HS and HS-glucose-mannitol media with an initial pH of 5.5 and 6.0, respectively. Similarly, a decreasing pH trend from 4.5 to 3.0 was reported by Nguyen et al. [17] during Kombucha fermentation using *Gluconacetobacter intermedius* KN89 and *D. bruxellensis* co-culture due to acid production. A pH reduction was also observed using *G. intermedius* SNT-1 in HS medium supplemented with pre-treated molasses as a carbon source [1].

**Figure 4:**
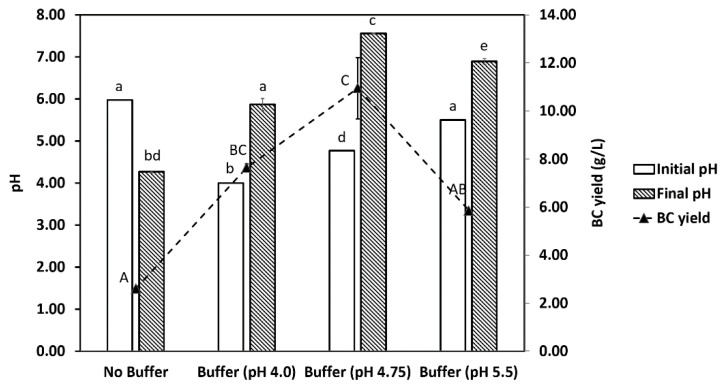
Effects of acetate buffer on pH and BC yield after 7 days of co-culture fermentation. Culture conditions: media composition, 150 g/L molasses, 500 mg/L caffeine, acetate buffer (no acetate buffer; 200 mM, pH 4.0, 4.75, 5.5); inoculum size, 10% *v*/*v*; *K. intermedius*: *D. bruxellensis* ratio, 1:1; temperature, 30 °C. Different upper-case letters indicate significant differences (*p* < 0.05) in BC yields.

The increasing trend in pH observed in this study could be attributed to sodium acetate in the acetate buffer used, as previously reported by Jeffery et al. [40]. The finding was confirmed in the present study as the pH decreased significantly (*p* < 0.05) from 5.98 to 4.27 within 7 days in the absence of acetate buffer (Figure 4). In contrast, a significant increase in pH was observed in its presence, regardless of the starting pH (4.0, 4.75, and 5.5). Nevertheless, the reason that acetate buffer causes the pH of molasses medium to rise warrants future investigations.

### 3.5. The Effect of Co-Culture on BC Production

BC production by *K. intermedius* in single and co-culture are shown in Figure 3c. Significant growth of the BC layer in both single and co-culture was observed during the first 3 days of fermentation. However, the production rate was higher in single culture (7.33 g/L/day) than in co-culture (5.5 g/L/day), producing 22 g/L and 16.5 g/L of BC by day 3, respectively (Table 2). After 3 days, BC growth in single culture reached a plateau, while it lasted until day 12 in co-culture. By the end of fermentation, the total BC yields obtained in single and co-culture were 22 g/L and 17.5 g/L, respectively. The results suggest that the presence of *D. bruxellensis* could cause a significant reduction (*p* < 0.05) in BC production (~24%) despite its stimulatory effect on *K. intermedius* growth (Figure 2a). In co-culture, *D. bruxellensis* might have utilized a significant portion of reducing sugar, limiting the amount of sugar available for *K. intermedius* to convert into BC. Another possible reason is that *D. bruxellensis* might have stimulated *K. intermedius* to convert a higher proportion of reducing sugar into acid production instead of BC, as previously reported by Nguyen et al. [17]. They suggest that *D. bruxellensis* can increase the level of acetic acid in the media that could provoke a feedback inhibition in the glycolysis pathway of *K. intermedius*, promoting glucose conversion into glucuronic acid. The two possibilities may explain why the pH during the early to mid-stage fermentation was more acidic in co-culture than in single culture (Figure 3c). Furthermore, the relatively lower pH in co-culture might provide a less favorable environment for *K. intermedius* to produce BC. The results shown in Figure 4 further confirm that the highest BC yield (13.19 g/L) was obtained when the pH increased to 7.56. In contrast, the lowest BC yield (2.60 g/L) was achieved when the pH dropped to 4.27. This result was in accordance with the study conducted by Lin et al. [21], which demonstrated the ability of *K. intermedius* FST213-1 to produce BC within the range of pH 4–9, with pH 8 resulting in the highest BC yield (1.2 g/L). Based on these results, it could be suggested that the BC production is more favorable in alkaline conditions, which in this case, could be achieved by adding acetate buffer into the media. However, the underlying mechanisms of how acetate buffer increases pH and eventually the BC yield require further investigation.

## 4. Conclusions

In this study, the effect of *D. bruxellensis* on *K. intermedius* growth and BC production was investigated. The results showed that *D. bruxellensis* could prolong *K. intermedius’* stationary phase up to 12 days and maintain a 2.43 log CFU/mL higher population by the end of the fermentation process. However, the BC production was compromised as the BC yield decreased by ~24% in co-culture. Moreover, an unusual increasing pH trend was observed as *K. intermedius* proliferated, associated with higher BC production. Although *K. intermedius* growth could be stimulated, further investigation should be conducted to find an optimum inoculation procedure, to prevent *D. bruxellensis* from interfering with the BC production.

## Figures and Tables

**Table 1:** Physicochemical characteristics of molasses used.

Parameter	Molasses
Colour	Brown/black
Brix, %	77.6
Sucrose, %	46.9
Inverted sugar, %	50.8
Purity, %	60.8
Specific gravity, %	1.4
pH	5.1
Ash conductivity, %	0.40

**Table 2:** Specific growth rate μ (h^−1^), the sugar consumption rate (g/L/d), initial production rate (g/L/d), and total BC yield (g/L) of *K.* *intermedius* (bacteria) and *D. bruxellensis* (yeast) in single and co-culture.

Sample	Specific Growth Rate μ (h^−1^) ^a^	Sugar Consumption Rate (g/L/day) ^b^	BC Production	
			Initial Production Rate (g/L/day) ^c^	Total Yield (g/L) ^d^
Single culture- *K. intermedius*	0.188 ± 0.01	15.9 ± 0.01	7.33 ± 0.01	22.0 ± 0.01
Single culture- *D. bruxellensis*	0.213 ± 0.18	25.8 ± 2.1	NA	NA
Co-culture:		46.4 ± 3.2	5.50 ± 0.5	17.5 ±1.5
*K. intermedius*	0.215 ± 0.01	NA	NA	NA
*D. bruxellensis*	0.223 ± 0.01	NA	NA	NA

^a,c^ Specific growth rate and initial BC production rate during the first 3 days of incubation. ^b^ Initial sugar consumption rate during the first 4 days of incubation. ^d^ Total yield produced after 15 days of incubation. Data measured for 10% inoculum size, NA: no assessment.
